# Identifying Plant-Based Natural Medicine against Oxidative Stress and Neurodegenerative Disorders

**DOI:** 10.1155/2020/8648742

**Published:** 2020-09-15

**Authors:** Rahul Chandran, Heidi Abrahamse

**Affiliations:** Laser Research Centre, Faculty of Health Sciences, University of Johannesburg, P.O. Box 17011, Doornfontein, 2028 Johannesburg, South Africa

## Abstract

Free radicals and oxidative stress are among the most studied factors leading to the imbalance in mental health. With no exception, free radicals also damage neuronal cells, leading to various degenerative diseases. With existing modern medications, around 80% of the world population relies on herbal medicine for various ailments. Phytochemicals in plants have a wide range of pharmacological properties, the major being their ability to scavenge free radicals. Plant polyphenols are among the major class of antioxidants identified in plants. This antioxidative property of plant compounds and their ability to downgrade the process of oxidative stress can be used to treat neurodegenerative diseases. However, selecting plants and their active compounds is a crucial step in framing the mechanism of action underlying their therapeutic potential.

## 1. Introduction

Herbal medicine and their active ingredients are trusted source of medicine since ancient times. Herbal products with plant parts in crude form or their bioactive compounds are gaining interest in the treatment of diseases [1]. Plant are rich in medicinal compounds and almost all the parts of a plant can be considered as a medicine in one way or another. However, the most commonly used parts are flowers, fruits, seeds, roots, leaves, bark, etc. Due to increasing disease forms, resistance to existing drugs and demand for drugs with lesser side effects, concern has raised to explore the best source of medicine with modern science/technology and ideas. Global pharmaceutical companies are in the run to find novel medicinal sources and plants being their best choice [2–4]. The popularity of herbal products has increased worldwide in the past few decades [5]. These days, herbal products with well-defined constituents are more preferred over crude forms due to their reliability in preclinical and clinical studies.

Depression and anxiety are among the most common neurodegenerative disorders and also highly associated with substantial comorbidity and mortality. Free radicals and oxidative stress might induce conditions pertaining to nervous disorders and behavioral changes [6]. Further, a better understanding of oxidative stress-induced mitochondrial dysfunction, neuroinflammatory response, and intracellular signaling pathways may help to draw up a relation among free radicals, oxidative stress, and neurodegerative disorders [7, 8].

Plant based-therapy targeting the relative link between oxidative stress and neurodegeration through cellular and molecular levels may improve strategies of treatment and drug development.

## 2. Oxidative Stress and Neurodegeneration

**Figure 1:**
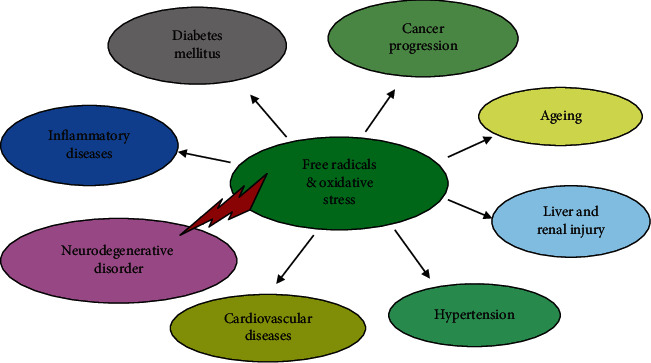
Pathogenesis of free radicals. Free radicals and oxidative stress are responsible for the development of various diseases through various cellular and molecular processes. Among them, neurodegeneraion is the most commonly noted disorder induced by free radicals.

Oxygen is an essential molecule, which during metabolic conditions may generate free radicals. Free radicals are also an essential and fundamental molecule in any biochemical process and are essential in redox reactions [9]. However, these radicals are highly unstable and target easily accessible biomolecules like lipids, nucleic acids, and proteins. This establishes a chain reaction and plays an important role in the pathogenesis of many disease conditions [10]. In recent years, the research community has witnessed new developments in free radical biology and their role in health and disease incidence (Figure 1). Compared to other organs, the brain uses a major portion of oxygen and has relatively less antioxidant enzymes, making them more prone to free radical attack [11]. Free radicals like reactive oxygen species (ROS) and reactive nitrogen species (RNS) are prime generators of stress.

**Figure 2:**
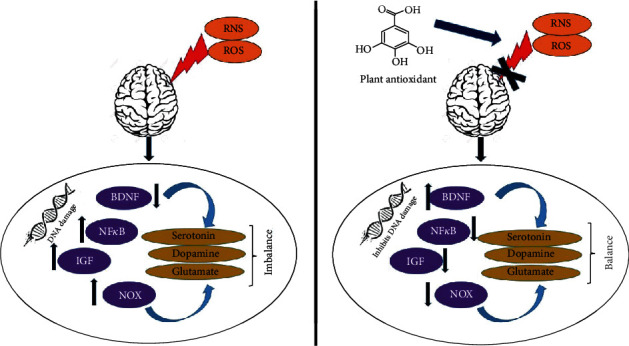
Antioxidant defense in neurodegenerative disorder. The radicals generated in the brain mitochondria cause damage to its DNA. Increased NADPH oxidase (NOX), nuclear factor *κ* B (NF*κ*B), and insulin-like growth factor (IGF) and low levels of brain-derived neurotrophic factor (BDNF) may cause imbalance in the neurotransmitter production. Antioxidant, on the other hand, reverses this action.

Due to high reactivity, superoxide radicals generated in the brain mitochondria limit their movement and cause damage to its DNA and lead to impaired function [12] and neurodegenerative diseases. Hydrogen peroxide (H_2_O_2_) is the major precursor of superoxide radical in mitochondria. Reduced nicotinamide adenine dinucleotide phosphate (NADPH) oxidase (NOX1 and NOX4) being another sources, are expressed in neurons [13]. Increased NOX activity in the microglia induces neuroinflammation [14] and neurodegenerative diseases [15]. The level of free radical damage increases when nitric oxide interacts with superoxide to become more toxic to neurons [16]. Brain-derived neurotrophic factor (BDNF) is one of the major factors determining the bipolar disorder and depression. Increased free radical concentration and oxidative stress are directly proportional to low concentrations of BDNF and promote depression and anxiety. The use of an antidepressant will increase BDNF, regenerate brain cells, and reduce oxidative stress, depression, and anxiety [17, 18]. Depression is characterized by mood fluctuation and short-term emotional changes leading to serious health. A similar mental disorder is observed in patients with anxiety showing symptoms such as insensitiveness, unpleasant feeling, and loss of interest [19]. The irregular production of neurotransmitters like serotonin, dopamine, and glutamate in the brain is also associated with neurodegeneration [20, 21]. Oxidative stress, increased levels of nuclear factor *κ* B (NF*κ*B) and insulin-like growth factor (IGF) is also linked with the progression of these disease conditions [22]. Oxidative stress and mechanisms leading to neurodegenerative and neuropsychiatric disorders have been well studied [23–26]. Antioxidants can remove these free radicals and suppress the conditions leading to depression and anxiety (Figure 2) [27–29].

## 3. Phytochemicals and Their Pharmacological Significance

According to the World Health Organization [30], around 5% of the population has anxiety and depression disorders. Plant medicine has shown wonders in the treatment of diseases, and traditional plant formulations have been well documented by many researchers [31–33]. Pharmacological reports using these traditional medicines are promising; the research on medicinal plants and neurological disorders are progressing worldwide [34]. With an extensive research on the biological and clinical aspects of depression and anxiety and existing side effects of synthetic drugs, it has become possible to offer new treatment strategies using herbal medicine. The clinical importance of a plant can be related to its biologically active compounds present in them. These compounds are produced in plants as primary and secondary metabolites for the defense mechanism against pathogens, abiotic stress, and other similar adverse conditions [35]. However, these phytochemicals are known to have therapeutic properties, provide nutrition for normal cell health and repairs, enhance the immune system, fight disease-causing agents, inhibit carcinogens, and act as antioxidants [36]. Some of them such as polyphenols, flavonoids, terpenoids, catechins, ascorbic acid, alpha-tocopherol, and beta-carotene may act as an effective nutraceutical supplement. Though their mechanism of actions is yet to be studied completely, their role in preventing the progression of neurodegenerative disorders including Parkinson's disease [37] and Alzheimer's disease are well evaluated [38]. These phytochemicals may exert their therapeutic effects as a single active compound or synergistically. Furthermore, additive action of crude extracts eliminates side effects associated with the predominance of a single xenobiotic compound, giving them a broad spectrum activity and reducing chances of developing resistance by pathogens [39].

**Figure 3:**
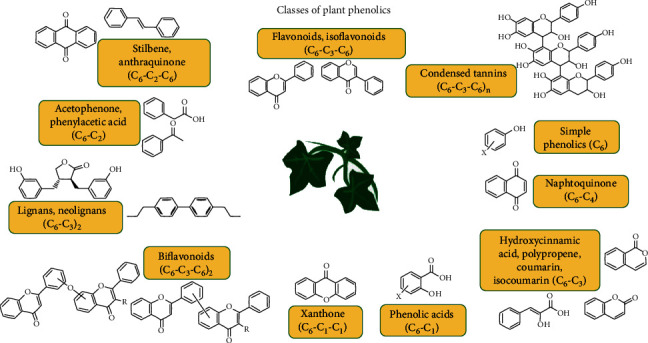
Classes of plant phenolics. The figure illustrates plant-derived compounds belonging to different classes of polyphenolics. These phenolic compounds have shown various forms of action to protect the brain from neurodegeneration.

Plants produce three major classes of phytochemicals, *viz.* phenolic metabolites and alkaloids, terpenoids, and other nitrogen-containing compounds [40].  Figure 3 represents major classes of plant-derived phenolic compounds. Among these, polyphenolics are well known for their high antioxidant capacity [41, 42]. Phenolics are compounds possessing one or more aromatic rings with one or more hydroxyl groups. Plant polyphenols, such as epicatechin, *β*-catechin, epicatechin gallate, epigallocatechin, tannic acid, isoflavones, glycyrrhizin, saponins, and chlorogenic acid, have an antidiabetic property [43]. Flavanoids have been reported to show therapeutic activity in cardiovascular diseases and atherosclerosis [44]. Basirnejad et al. [45] reviewed the protective role of carotenoids including lycopene, *γ*-carotene, lutein, and xanthophyll against cancer progression. In animals, phytosterols exhibit anti-inflammatory, antineoplastic, antipyretic, and immune-modulating activities [46]. Flavonoids perform a wide range of actions against free radical-mediated inflammation, tumors, and cellular signaling, [47]. These impaired signaling may cause neurodegenerative disorders.

Researchers have also reported neurotrophin induction properties of plant-derived natural compounds. Phytocompounds like 3,5-dicaffeoyl-mucoquinic acid *(Aster scaber)*, furostanol saponins, diosgenin, diosniposide A-B, diosniponol C-D, (*Dioscorea* spp.), 6-shogaol (*Zingiber officinale*), and lignans (*Abies holophylla*) were found to possess nerve growth factor (NGF) mimicking property. Other compounds like 3,7-dihydroxy-2,4,6-trimethoxy-phenanthrene, ginkgolide B, lignan derivatives, 4,6-dimethoxyphenanthrene-2,3,7-triol, spicatoside A, ginsenoside Rg3, quercetin, apigenin derivatives, cyanidin-3-*O*-*β*- glucopyranoside, quinic acid derivatives, and clerodane diterpenoids have been well reported in inducing neuronal cell differentiation and upregulating BDNF [48, 49]. Plant parts as a raw material in traditional medicine or their defined active compounds in modern natural medicine are an interesting source of treatment against neurodegenerative disorders (Table 1).

## 4. Selection of Plant

There are certain strategies used for the selection of plant species: random screening and ethnobotany. With over 500,000 plant species on earth, and each of these with flowers, fruits, leaves, stem, bark, and roots with different chemical compositions, geographical and seasonal, the likelihood of finding an appropriate plant sample for a desired disease through random search is fairly difficult [60, 61]. In some cases, the compounds isolated from such plants may not be novel and show good activity compared to those available in the market. However, a plant with its ethnobotanical background could be selected with the belief that it is being used traditionally with some medicinal purpose. Traditional knowledge also includes detail of the season during which a particular plant species is medicinally active, part of the plant used, geographical region in which a species abundant [62–64]. Long-term use of a medicinal plant in traditional medicines, including folklore remedies, is generally considered safe and active against prevention of many diseases, and has been proven to be a trustworthy source of active compounds. Such a correlation between traditional medicine and their use in research and the isolation of compounds are well studied by many researchers [65–67].

## 5. Extraction and Isolation of Bioactive Compounds

As discussed, plant polyphenolics are the major class of antioxidants, which are widely studied for their disease prevention, free radical scavenging property, and reducing oxidative stress. Extraction of these and other compounds is the crucial step in the analysis of plants for its medicinal property. It is necessary to extract the desired chemical components from the plant materials for further separation and characterization. Extraction and isolation of active compounds from plants are tedious processes. Hence, definitive measures must be taken to restore the bioactive compounds while extraction and to assure that they are not destroyed, lost, or distorted. It is important to follow traditional uses of a medicinal plant and prepare an extract to mimic as closely as possible the traditional ‘herbal' drug [68]. The selection of a solvent system largely depends on the specific nature of the bioactive compound being targeted. Different solvent systems are available to extract the bioactive compound from natural products. As the target compounds may vary from polar to nonpolar, the suitability of the methods of extraction must be considered. Various methods, such as sonication, Soxhlet extraction, heating under reflux, and others are commonly used [69–71] for the extraction of compounds from plant samples. In addition, maceration or percolation of fresh green plants or dried powdered plant material in water and/or organic solvent systems is also being used. In order to reduce the consumption of solvents and time, several modern techniques have been introduced. These include solid-phase microextraction, supercritical-fluid extraction, pressurized-liquid extraction, microwave-assisted extraction, solid-phase extraction, and surfactant-mediated techniques, which possess certain advantages [72]. These steps improve extraction efficiency, kinetics of extraction, and selectivity [73]. But the conventional methods like Soxhlet and maceration are still under use due to their high efficiency in extracting the phytocompounds with higher extract yield. However, the wide range of compounds in plants makes separation and isolation of unknown active compounds a difficult ask. Activity-guided fractionation is the most frequently used technique for isolating plant compounds [74]. The optimum recovery of antioxidant compounds like polyphenolics is different from one sample to the other and relies on the type of plant used. The choice of extraction solvents such as ethyl acetate, acetone, alcohols (methanol, ethanol, and propanol), water and their mixtures [75] will influence the yields of phenolics extracted.

According to research findings, increasing time and temperature will increase the solubility and extractability of compounds; however, plant phenolics may undergo enzymatic oxidation and reactions forming undesirable compounds under such conditions [76, 77]. Sample matrix and particle size also strongly influence phenolic extraction from plant materials [78]. Flavonoids are often extracted with methanol, ethanol, acetone, water, or mixtures of these solvents using heated reflux extraction methods [79–81]. Similarly, maximum extractability of flavonoids can be achieved using polar organic solvents alone or in combination [82, 83].

Maceration, Soxhlet, and heated reflux extraction are simple, require relatively cheap apparatus, and result in adequately high phenolic extraction rates [84, 85]. However, the need for large volumes of hazardous organic solvents, long extraction times, and degradation of targeted components due to air, light, high temperatures, and enzymatic reactions are few noted disadvantages [86, 87] which needs standardization. Other modern techniques include pressurized liquid extraction (PLE), super critical fluid extraction (SFC), and microwave-assisted extraction (MAE) [74].

## 6. Purification and Structural Determination

**Figure 4:**
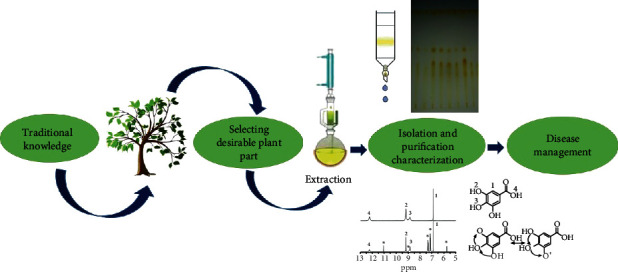
Process of activity-guided isolation of compounds. Based on the traditional knowledge, plants were collected and processed for extraction. Extraction and solvent systems are influenced by the plant part, desired activity, targeted class of compounds, etc. Finally, the purified compound alone or in combination as crude extract is used in disease management.

Purification of the phytocompounds from the crude extract is a difficult and crucial part. Advances in modern techniques of isolation and purification have opened up possibilities for large-scale production of active compounds from plants [78]. Based on the solvent system and techniques used, the crude extract displays combination of bioactive compounds or phytochemicals with different polarities. Chromatographic separations are the best used techniques implemented for efficient isolation and purification of targeted phytocompounds. Paper, thin layer, and column chromatography are the best used conventional isolation and purification method to achieve maximum yield [88, 89]. Finally, the structural determination of compounds after isolation can be done by accumulating data from a wide range of spectroscopic techniques, such as UV-visible, infrared (IR), and nuclear magnetic resonance (NMR) spectroscopy (Figure 4). Although almost all parts of the electromagnetic spectrum are used for studying matter in organic chemistry, but natural products are concerned with energy absorption from three or four regions—ultraviolet (UV), visible, infrared (IR), radio frequency, and electron beam [90].

In clinical trials, isolating active principle and their use are frequently investigated compared to crude extract in order to determine the exact mechanism of action. However, the combination of various active principles of crude extracts promotes synergistic effects, leading to an antioxidant-based defense mechanism in patients with neurodegeneration [91]. Moreover, natural antioxidants with multitarget drug profiles can suppress oxidative stress and the combination of single active principle or crude extracts needs further investigation.

## 7. Conclusion and Future Perspective

In conclusion, free radicals and oxidative stress could act as one of the prime precursors in building up neurodegenerative disorders. Phytochemicals with a broad range of activities has increased interest among researchers to explore plant species with significant traditional use. Moreover, it is important to note that natural products, especially from plants with antioxidant property can present a reliable source of medicine. The process of identifying plants and using them for desired medication is a tedious process. But, once identified, herbal medicine could noticeably have maximum impact with lesser side effects due to the synergistic action of compounds present in them. However, more research is needed in this direction to justify their use in the disease management.

## Figures and Tables

**Table 1:** Commonly used plants against neurodegenerative disorders.

Plant name	Parts used	Active compound	Action	References
*Ginkgo biloba* L	Leaves	Quercetin, kaempferol, and isorhamnetin	Improves cerebral blood flow	[50]
*Panax ginseng* C.A. Meyer	Arial parts and root	Aglycones, protopanaxadiol, and propanaxatriol	Promotes neuron survival, increasing the levels of neurotrophic factors	[51]
*Scutellaria baicalensis* Georgi	Arial parts and root	Baicalein, baicalin, and wogonin	Protect neurons from oxidative damage	[52]
*Curcuma longa* L	Rhizome	Curcumin	Inhibition of cytokine production and microglia activation	[53]
*Vitis vinifera* L.	Fruits and seeds	Resveratrol, quercetin, and catechin	Neuroprotective effects	[54]
*Salvia officinalis* L.	Leaves and flowers	1,8-Cineole, camphor, borneol, caryophyllene, and linalool	Anticholinesterase activity	[55]
*Coffea* spp.	Seeds	Caffeine	Acts on adenosine receptors	[56]
*Camellia sinensis* Kuntze	Leaves	Epigallocatechin, epigallocatechin-3-gallate, myricetin, quercetin, kaempherol, epicatechin	Antioxidants, protects from oxidative stress, reduces amyloid proteins	[57]
*Bacopa monniera* (L.) Pennel	Whole plant	Herpestine, d-mannitol, hersaponin, and monnierin	Enhancing neuronal synthesis, kinase activity, restoration of synaptic activity, and nerve impulse transmission	[58]
*Centella asiatica* (L.) urban	Leaves	Asiaticoside, brahmoside, brahminoside, asiatic acid, madecassic acid, brahmic acid, isobrahmic acid, and betulic acid	Antioxidant action, acetylcholine esterase inhibitor activity	[59]
*Picrorhiza scrophulariiflora* Pennell	Roots	Glycosides, terpenoids, phenylethanoid, glycosides, and phenolic glycosides	Neuritogenic activity	[58]

## Data Availability

All the data used in this study are available with the corresponding author upon request.
